# Neutrophils from Patients with Primary Ciliary Dyskinesia Display Reduced Chemotaxis to CXCR2 Ligands

**DOI:** 10.3389/fimmu.2017.01126

**Published:** 2017-09-22

**Authors:** Maaike Cockx, Mieke Gouwy, Véronique Godding, Kris De Boeck, Jo Van Damme, Mieke Boon, Sofie Struyf

**Affiliations:** ^1^Laboratory of Molecular Immunology, Department of Microbiology and Immunology, Rega Institute for Medical Research, University of Leuven, Leuven, Belgium; ^2^Unité de Pneumologie Pédiatrique et Mucoviscidose, Clinique Universitaire Saint-Luc UCL Brussels, Brussels, Belgium; ^3^Pediatric Pulmonology and Cystic Fibrosis Unit, Department of Pediatrics, University Hospitals Leuven, Leuven, Belgium

**Keywords:** chemokines, chemotaxis, cytokines, IL-1, inflammation, neutrophils, pulmonary disease

## Abstract

Primary ciliary dyskinesia (PCD), cystic fibrosis (CF), and chronic obstructive airway disease are characterized by neutrophilic inflammation in the lungs. In CF and chronic obstructive airway disease, improper functioning of neutrophils has been demonstrated. We hypothesized that the pulmonary damage in PCD might be aggravated by abnormal functioning neutrophils either as a primary consequence of the PCD mutation or secondary to chronic inflammation. We analyzed chemotactic responses and chemoattractant receptor expression profiles of peripheral blood neutrophils from 36 patients with PCD, 21 healthy children and 19 healthy adults. We stimulated peripheral blood monocytes from patients and healthy controls and measured CXCL8 and IL-1β production with ELISA. PCD neutrophils displayed reduced migration toward CXCR2 ligands (CXCL5 and CXCL8) in the shape change, microchamber and microslide chemotaxis assays, whereas leukotriene B4 and complement component 5a chemotactic responses were not significantly different. The reduced response to CXCL8 was observed in all subgroups of patients with PCD (displaying either normal ultrastructure, dynein abnormalities or central pair deficiencies) and correlated with lung function. CXCR2 was downregulated in about 65% of the PCD patients, suggestive for additional mechanisms causing CXCR2 impairment. After treatment with the TLR ligands lipopolysaccharide and peptidoglycan, PCD monocytes produced more CXCL8 and IL-1β compared to controls. Moreover, PCD monocytes also responded stronger to IL-1β stimulation in terms of CXCL8 production. In conclusion, we revealed a potential link between CXCR2 and its ligand CXCL8 and the pathogenesis of PCD.

## Introduction

Primary ciliary dyskinesia (PCD) is a rare genetic disease, caused by abnormal structure and/or function of motile cilia (1, 2). Patients suffer from recurrent pulmonary infections that cause a decline in lung function over time, chronic upper airway symptoms, and almost half have laterality defects. Infections are the result of ineffective mucociliary clearance and could lead to a chronic and non-resolving activation of the innate immune system. Particularly, neutrophilic granulocytes are abundantly present in PCD lungs (3).

In other chronic airway diseases characterized by neutrophilic infiltrates, improper functioning of those phagocytes has been demonstrated (4, 5). The pulmonary damage in PCD might also be aggravated by abnormal functioning neutrophilic granulocytes. Indeed, in some old reports, the migratory capacity of peripheral blood neutrophils from patients with PCD (PCD PMN) toward *N*-formylmethionyl-leucyl-phenylalanine (fMLP), leukotriene B4 (LTB4) and complement component 5a (C5a) has been shown to be reduced (6, 7), whereas others reported unaffected chemotaxis of PCD PMN to the bacterial peptide fMLP or rather aspecific stimuli, such as zymosan (8, 9).

Meanwhile, the various molecules and pathways contributing to the different steps in PMN extravasation and infiltration into inflamed lungs have been better characterized (10). In this study, we investigated chemotactic responses of PCD PMN to the CXC chemokines CXCL5 and CXCL8 that both activate the chemokine receptor CXCR2. In addition, CXCL8 functions through CXCR1 as well, which is co-expressed on PMN with CXCR2 and CXCR4. We also tested LTB4 and C5a as chemotaxis inducers, which similar to chemokines, activate G protein-coupled receptors to guide phagocytes to infected tissues. We evaluated migration of PMN from patients with PCD and healthy children and adults in the Neuro Probe microchamber chemotaxis assay. Afterward, PMN from patients that showed decreased migration in the Neuro Probe chemotaxis assay were studied in μ-slide chemotaxis and shape change assays. Furthermore, we evaluated whether PCD PMN have an aberrant chemoattractant receptor expression profile compared to healthy individuals. Finally, we measured CXCL8 production after treatment of PCD monocytes with inflammatory stimuli.

## Materials and Methods

### Patients and Healthy Controls

Thirty-six patients with PCD (range 2–26 years, average age 13 years; for details, see Table 1) were enrolled between 2012 and 2016 at the university hospitals of KU Leuven. Patients were only included when they were clinically stable (no change in cough or sputum, no fever, no change in therapy for a period of at least 2 weeks, change in FEV_1_ < 10% since the last measurement). The study protocol (S57236[ML11095]) was approved by the ethical committee of the university. All patients underwent a combination of diagnostic testing: all had abnormal ciliary activity after cell culture of nasal biopsies. Electron microscopy of the cilia showed outer dynein arm deficiency (Dynein) in 16, microtubular disarrangement with inner dynein deficiency and central pair abnormalities (CP) in 1, absence of the central pair in 2, ciliary aplasia in 1, and normal ultrastructure (NU) in 16 patients. Genetic analysis confirmed disease causing mutations in 24 patients, did not provide evidence for mutations in seven patients and was not performed in five patients.

**Table 1:** Patient characteristics.

Patient	TEM abnormality	Genetic defect	*Situs inversus*	Bronchiectasis	WBC (/μl)	CRP (mg/dl)	FVC (% pred)	FEV_1_ (% pred)	Sputum culture	Chronic infection	Antibiotics or Ig	Azithromycin	Inhaled steroids
1	NU	DNAH11	0	1	7,020	<0.3	97	91	–	–	–	–	–
2	NU	DNAH11	1	1	7,650	<0.3	114	98	–	–	–, iv immunoglobulins	+	+
3	NU	DNAH11	0	1	6,420	4.9	78	90	–	–	–, iv immunoglobulins	+	+
4	ODA	DNAH5	0	1	6,840	2.4	96	76	–	–	–	–	+
5	CP + IDA	CCDC40	0	1	4,640	0.3	66	38	–	–	+	–	+
6	ODA	*ND*	0	1	*ND*	*ND*	96	88	*H. influenzae*	–	–	+	+
7	NU	CCDC65	0	1	*ND*	1.1	65	38	*H. influenzae*	*H. influenzae*	–	+	+
8	ODA	DNAH5	1	1	9,880	1.9	73	64	*P. aeruginosa*	*P. aeruginosa*	–	+	+
9	ODA	DNAAF1	0	0	9,500	<0.3	112	110	*S. aureus*	–	–	–	–
10	ODA	DNAAF1	0	0	9,500	0.6	121	108	–	–	–	–	+
11	ODA	DNAH5	0	1	9,140	1.8	122	111	*S. aureus*	–	–	–	–
12	ODA	*ND*	0	0	6,160	<0.3	74	74	–	–	+	–	–
13	ODA	*ND*	1	1	8,300	0.6	82	82	*H. influenzae*	–	–	–	–
14	CP	RSPH4	0	*ND*	5,100	0.3	94	94					
15	ODA	*ND*	0	1	4,700	<0.3	78	78	*H. influenzae*	–	–, subcutaneous immunoglobulins	+	+
16	ODA	DNAH5	0	1	7,380	1.6	103	103	*S. pneumoniae*	–	+	–	+
17	NU	*ND*	0	1	5,100	2.1	71	71	*S. aureus, M. catarrhalis*	–	–	–	–
18	NU	DNAH11	0	1	5,990		110	110	*ND*	–	–	+	–
19	NU	DNAH11	1	1	9,540	0.3	82	82	–	–	–	+	–
20	NU	DNAH11	0	0	7,570	0.3	*ND*	*ND*	*P. aeruginosa, H. influenzae*	–	+	–	–
21	NU	DNAH11	1	1	6,080	2.4	93	93	–	–	–	–	–
22	ODA	CCDC103	1	0	6,880	1.1	108	108	–	–	–	–	–
23	ODA	*ND*	1	0	9,220	2	98	98	*H. influenzae*	*H. influenzae*	–	–	–
24	ODA	DNAH5	1	0	17,240	1	113	113	–	–	–	–	+
25	NU	DNAH11	0	1	7,300	0.3	108	108	–	–	–	+	+
26	NU	HYDIN	0	1	6,310	3.2	72	72	*S. aureus, M. catarrhalis, H. influenzae*	*H. influenzae*	–	–	–
27	NU	DNAH11	0	1	7,300	<0.3	107	101	*ND*	*ND*	–	+	+
28	NU	CCDC103	1	0	7,350	<0.3	*ND*	*ND*	*ND*	–	+	–	–
29	CP	*ND*	0	1	*ND*	*ND*	110	112	*S. aureus*	*ND*	*ND*	*ND*	*ND*
30	NU	*ND*	0	0	*ND*	*ND*	104	97	–	*ND*	*ND*	*ND*	*ND*
31	ODA	ARMC4	1	1	*ND*	*ND*	98	74	–	*ND*	*ND*	*ND*	*ND*
32	NU	*ND*	1	1	*ND*	*ND*	75	71	–	*ND*	*ND*	*ND*	*ND*
33	NU	DNAH11	1	1	*ND*	*ND*	86	91	*S. aureus*	*ND*	*ND*	*ND*	*ND*
34	Ciliary aplasia	*ND*	0	0	*ND*	*ND*	88	84	*P. aeruginosa*	*ND*	*ND*	*ND*	*ND*
35	ODA	*ND*	1	0	*ND*	*ND*	98	90	–	*ND*	*ND*	*ND*	*ND*
36	ODA	*ND*	0	0	*ND*	*ND*	110	112	–	*ND*	*ND*	*ND*	*ND*

*Ultrastructural and genetic defects, clinical presentation (*situs inversus*, bronchiectasis, WBC (/μl) and CRP (mg/dl) concentrations in blood circulation, FVC (% pred), FEV_1_ (% pred), bacteria strains (*Haemofilus influenzae, Moraxella catarrhalis, Pseudomonas aeruginosa, Staphylococcus aureus*, and *Streptococcus pneumoniae*) isolated from sputum and chronic infection in airways) and applied treatment (antibiotics, azithromycin, immunoglobulins, and inhaled steroids) of the studied patients with PCD*.

*CP, central pair; CRP, C-reactive protein; FEV_1_ (% pred), forced expiratory volume in 1 s (predicted%); FVC (% pred), forced vital capacity (predicted%); IDA, inner dynein arm; Ig, immunoglobulins; ND, not determined; NU, normal ultrastructure; ODA, outer dynein arm; TEM, transmission electron microscopy; WBC, white blood cell count*.

Because neutrophil migration needs to be performed with freshly isolated cells and because maximally two patients could be evaluated on the same day, we included in each experiment PMN from a healthy adult control. This study, thus, included two control groups: a pediatric control group (*n* = 21; range 4–18 years, average age 10 years) that corresponded best to the enrolled patients and an adult control group (*n* = 19; range 23–41 years; average age 29) because it was impossible to organize blood donations of healthy children and children with PCD on the same day. Healthy pediatric controls were included as there are no available data on pediatric values of neutrophil function and expression of receptors in function of age. The healthy controls (or their parents) all signed informed consent and their blood samples were processed identically to those of the patients (same transport time, conditions, and handling time).

### Cell Isolation

Blood samples were collected in EDTA^+^ tubes. Peripheral blood granulocytes and peripheral blood mononuclear cells (PBMCs) were separated and isolated from whole blood by density gradient centrifugation as described (11). First, the blood sample was diluted with D-PBS (Lonza, Belgium) and gently layered on Ficoll-sodium diatrizoate (Lymfoprep, Axis-Shield PoC AS, Oslo, Norway). After centrifugation (400 × *g*, 30 min, 20°C, without break), PBMCs and plasma were separated (top layers) from the granulocytes and red blood cells (bottom layer). After collection, PBMCs were washed twice, counted in a hemocytometer and were then ready for use. The red pellet of neutrophils and erythrocytes was gently mixed with a starch solution (6% Dextran; Sigma) and the mixture was incubated for 30 min at 37°C to cause aggregation and sedimentation of the red blood cells. After a wash step with D-PBS, the residual red blood cells in the granulocyte preparation were removed by performing a hypotonic shock. After two additional wash steps, the neutrophilic granulocytes (>95% of the total granulocytic fraction) were counted in a hemocytometer and were then ready for use.

### 48-Well Micro Chamber Chemotaxis Assay

Neutrophil migration toward CXCL5, CXCL8, LTB4, and C5a was measured in a 48-well micro chemotaxis chamber (Neuro Probe, Gaithersburg, MD, USA) as described (12). LTB4 and CXCL8 (72 AA) were bought from Peprotech (Rocky Hill, NY, USA), complement component C5a from Sigma (St. Louis, MO, USA) and CXCL5 from R&D Systems (Abingdon, UK). In every assay, a reference adult control (healthy member of the laboratory staff) was included in order to allow normalization. Migration of the PCD PMN was expressed relative to migration of the reference adult control. The same approach (i.e., standardization to the healthy adult control) was used to study the migration of PMN from healthy children. Before we tested the migration of neutrophils from patients with PCD, we first tested dose–response curves of healthy neutrophils to determine the optimal concentration of chemoattractant to be tested with patient cells.

### Flow Cytometry

Flow cytometry was used to compare the expression of chemoattractant receptors on PMNs of patients with PCD and healthy controls. PMNs {3 × 10^5^ cells, diluted in FACS buffer [D-PBS + 2% Fetal Calf Serum (FCS)]} were stained with the following monoclonal antibodies: unlabeled anti-CXCR1, anti-CXCR2, and anti-C5aR; PE-labeled anti-CD16 and anti-BLT1. The cells were incubated with the antibodies for 30 min and afterward washed three times with FACS buffer. Cells incubated with unlabeled antibodies were subsequently stained with goat anti-mouse PE-labeled antibody and again incubated for 30 min on ice. After three additional washing steps, the cells were fixed with FACS buffer containing 0.4% paraformaldehyde. Cell suspensions were applied to a FACSCalibur flow cytometer (BD Biosciences) and the results were analyzed by CellQuest software (BD Biosciences). The FSC/SSC gate settings for PMN were verified with anti-CD16 antibodies.

### Shape Change Assays

The morphological shape changes that PMNs rapidly undergo when stimulated with chemotactic stimuli were examined as described (12). 50 µl of shape change buffer (HBSS without calcium and magnesium; supplemented with 10 mM HEPES) or 50 µl in shape change buffer diluted chemokine was added in duplicate to a 96-well plate. Shape change buffer was used as negative control. After adding the PMNs to the plate (3 × 10^4^ cells/50 μl), the cells were fixed with 100 µl/well 4% paraformaldehyde in shape change buffer at time points 0 and 1 min. For each condition, at least 200 cells/stimulus were microscopically (200× magnification) evaluated by an additional independent researcher who was blinded for the experimental conditions. We calculated the percentage of non-activated (perfectly round) cells and activated cells (showing cellular extensions and an irregular cell shape). Before we tested the activation of neutrophils from patients with PCD, we first tested dose–response curves of healthy neutrophils to determine the optimal concentration of CXCL5 and CXCL8 to be tested with patient cells.

### μ-Slide 2D Chemotaxis Assay

*Via* time-lapse microscopy, the μ-slide chemotaxis assay allows to study directionality, velocity, and total distance covered during the migration of PMNs toward a concentration gradient of chemokines (13). A microfluidic chamber (μ-slide VI, IBIDI, München, Germany) was used to create a stable concentration gradient of CXCL8. PMNs of patients with PCD and adult controls (3 × 10^6^ cells/ml) were suspended in RPMI 1640 + 2 mM HEPES + 0.5% HSA (μ-slide chemotaxis buffer) and after injection of the cells in the channel, the microfluidic chamber was incubated at 37°C for 30 min to allow the PMNs to settle down. Perpendicular on the channel with the cells, a concentration gradient of CXCL8 (200 ng/ml in μ-slide chemotaxis buffer) was created. Every 90 s, a snapshot of the cells was made with an inverted microscope (10× phase-contrast objective; Zeiss Axiovert 200 M) for 2 h. Constant temperature (37°C) and CO_2_ concentration (5%) were maintained throughout the recording. Migration of 20 randomly picked PMNs of each donor was tracked with the ImageJ manual tracking plug-in and data were analyzed with the IBIDI chemotaxis and migration tool. The optimal concentration of CXCL8 was determined in pilot experiments with healthy neutrophils.

### Induction Experiments and CXCL8 Measurements

Freshly isolated PBMCs (containing both lymphocytes and monocytes) were diluted in induction medium (2 × 10^6^ c/ml; RPMI 1640 + 2% FBS + 0.01% gentamycin) and seeded in 48-well plates. Cells were stimulated with 500 ng/ml lipopolysaccharide (LPS), 10 µg/ml peptidoglycan (PGN) or 100 ng/ml recombinant human IL-1β at 37°C and 5% CO_2_. After 24 h, the cell supernatants were collected and stored at −20°C. CXCL8 (14) and IL-1β (R&D Systems) concentrations in the cell supernatants, and known to be mainly produced by the monocytes, were determined by ELISA (detection limit 10 pg/ml CXCL8 and 5 pg/ml IL-1β). The IL-1β Duoset ELISA principally measures the active cytokine and is only marginally cross-reactive with pro-IL-1β, according to the manufacturer.

### Statistical Analysis

Normal distribution of the data was verified by the D’Agostino & Pearson normality test. Since the results were not normally distributed, non-parametric statistical tests were performed. First, non-parametric one-way ANOVA (Kruskal–Wallis test) was performed and afterward pairwise comparisons (Mann–Whitney *U* test) were performed to detect statistical differences between two groups using GraphPad software (GraphPad Software Inc., La Jolla, CA, USA). Significant differences detected by the Mann–Whitney *U* test are indicated on the figures and in the text. The chi-square test was applied to test whether receptor expression levels were more often reduced in patients compared to controls. Finally, Pearson correlation analysis was executed to assess a possible correlation between CXCR2 expression levels or CXCL8 response and lung function. A *p*-value <0.05 was regarded statistically significant.

## Results

### Peripheral Blood Neutrophils from Patients with PCD Display Reduced Chemotactic Responses to CXCR2 Ligands

**Figure 1:**
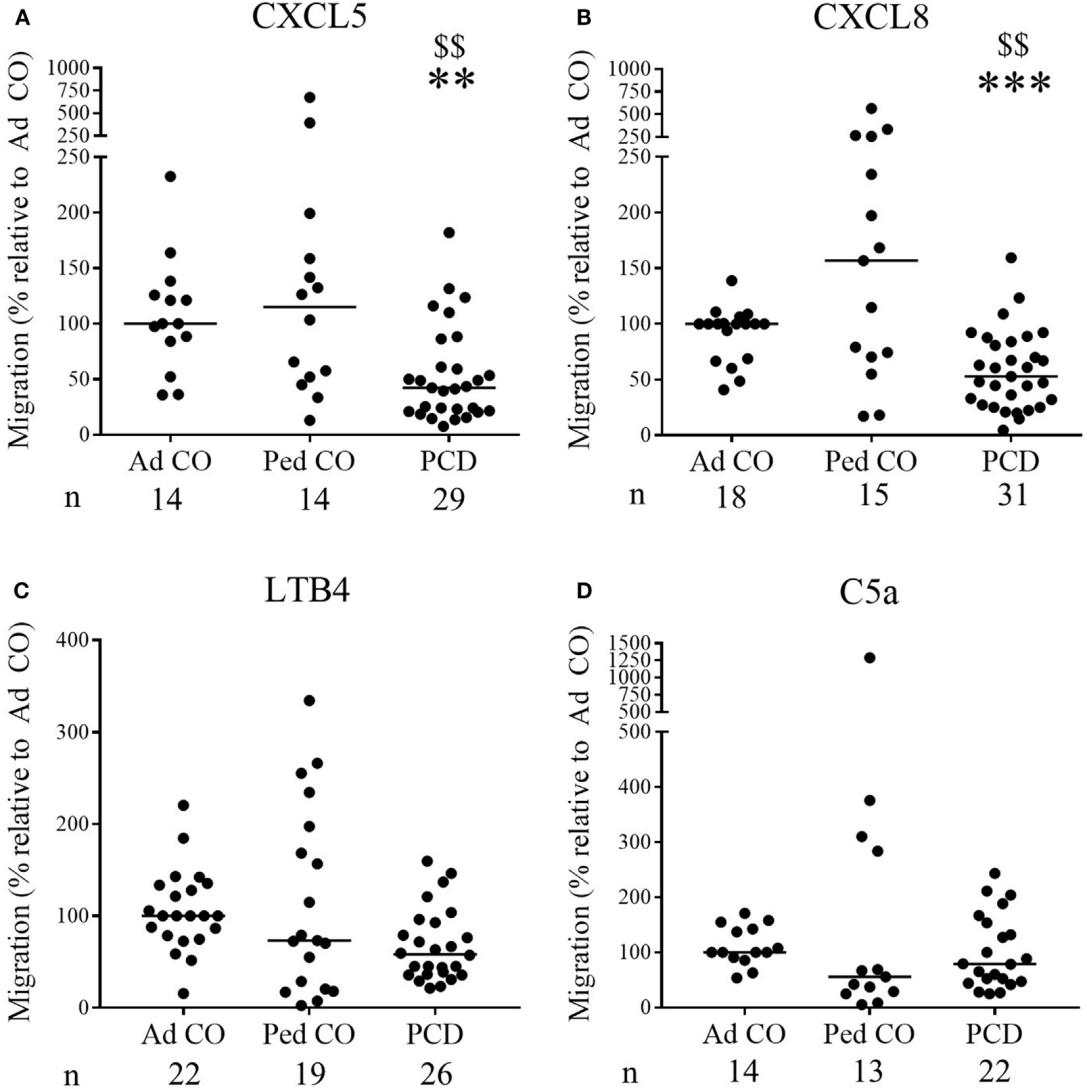
The chemotactic responses of peripheral blood PMN from patients with primary ciliary dyskinesia (PCD) are significantly lower to CXCL5 and CXCL8, but are normal to leukotriene B4 (LTB4) and complement component 5a (C5a). PMN from patients with PCD, healthy children (Ped CO) and healthy adults (Ad CO) were added to Neuro Probe chemotaxis chambers to compare their migratory capacity. The chemotactic index (CI; average number of migrated cells in response to the chemoattractant divided by the average number of spontaneously migrated cells) was used to express the chemotactic response. The chemotaxis toward **(A)** CXCL5 (10 ng/ml), **(B)** CXCL8 (10 ng/ml), **(C)** LTB4 (10^−8^ M), or **(D)** complement component C5a (10 ng/ml) was expressed relative to the chemotactic potency of the PMN from the reference Ad CO that was included in each Neuro Probe chemotaxis chamber on the same day (%). Each dot represents a separate blood donor and the horizontal lines indicate the median value. The number of blood donors (*n*) in each experimental group is indicated below the *x*-axis of a figure. Statistical data analysis was performed using the Mann–Whitney *U* test (Ad CO versus PCD: **p < 0.01, ***p < 0.001; Ped CO versus PCD: ^$$^*p* < 0.01).

**Figure 2:**
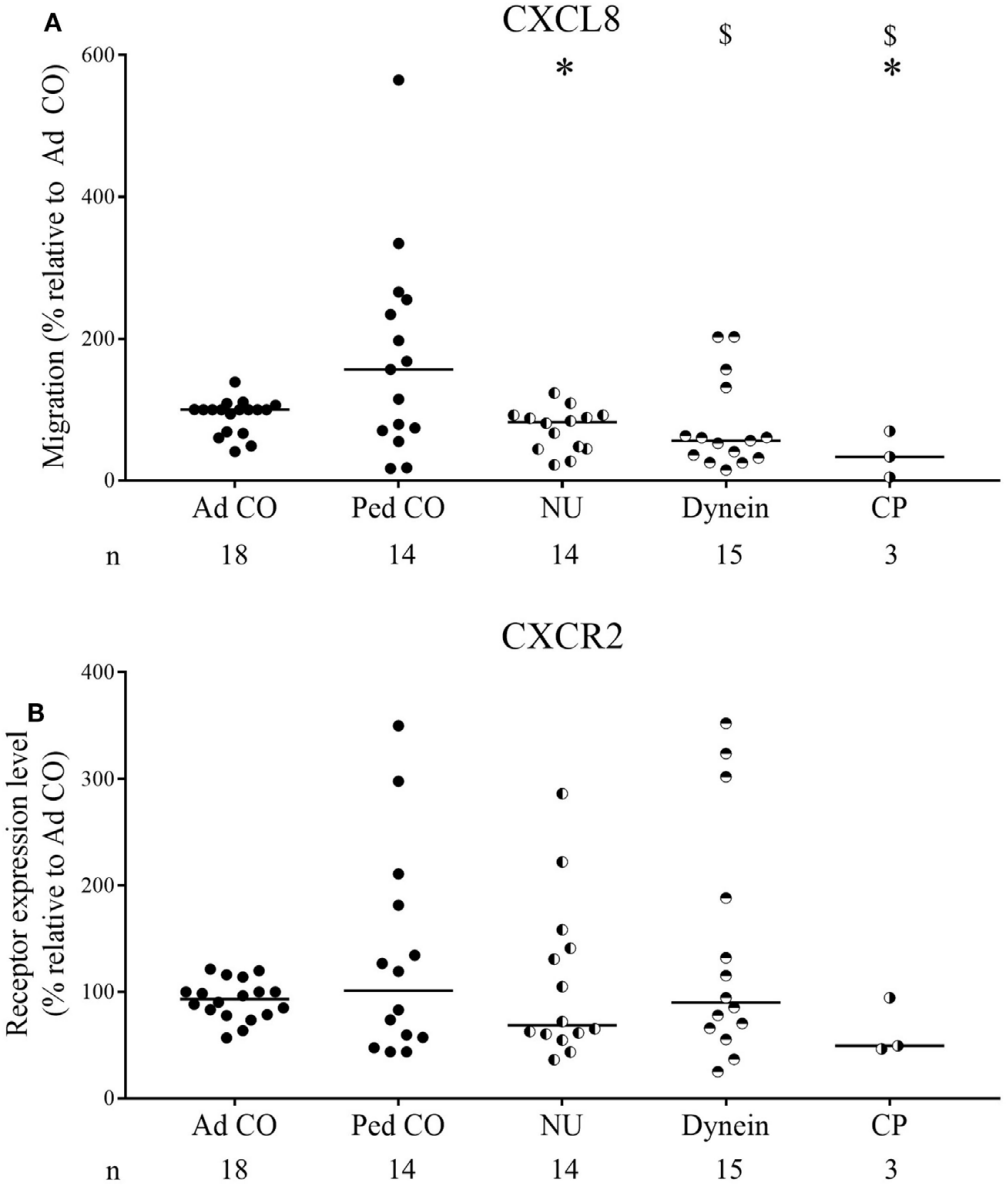
Chemotactic responses and chemoattractant receptor expression levels of primary ciliary dyskinesia (PCD) PMN following classification according to the diagnosed ultrastructural cilium defect. The obtained results of the chemotactic responses and CXCR2 expression levels were grouped for the three subclasses of patients: patients with normal ultrastructure (NU), dynein arm deficiencies (Dynein), and central pair abnormalities (CP) to detect a correlation between decreased migration and a certain ultrastructural cilium defect. The chemotactic response to CXCL8 of PMN from patients with PCD, healthy children (Ped CO) and healthy adults (Ad CO) was assessed in Neuro Probe chemotaxis chambers **(A)**, while simultaneously the expression levels of CXCR2 were measured by flow cytometry **(B)**. The chemoattractant receptor expression levels (mean fluorescence intensity) were normalized to the levels on PMN from the reference Ad CO (%). Each dot represents a separate blood donor and the horizontal lines indicate the median value. The number of blood donors (*n*) in each experimental group is indicated below the *x*-axis. Statistical data analysis was performed using the Mann–Whitney *U* test (Ad CO versus PCD: *p < 0.05; Ped CO versus PCD: $ *p* < 0.05).

Increased CXCL8 levels were previously detected in PCD sputum (3, 15), but migration of PCD PMN toward CXC chemokines has not yet been investigated. Although PMN from some patients with PCD displayed increased migratory capacity in the Neuro Probe microchamber chemotaxis assay, overall PCD PMN showed reduced migration toward CXCL5 and CXCL8 (Figures 1A,B, respectively) compared to healthy PMN (*p* = 0.0011 and *p* = 0.0003, respectively), whereas migration toward LTB4 and C5a was not altered (Figures 1C,D, respectively). Non-normalized chemotaxis results for CXCL5 and C5a are shown in Figure S1 in Supplementary Material. In order to detect whether reduced chemokine-induced migration only occurred in a subset of patients, chemotactic responses to CXCL8 and CXCR2 expression levels were displayed according to the structural cilium characteristics (NU, versus Dynein, versus CP) (Figure 2). Figure 2A shows that decreased migration to CXCL8 is observed in all subgroups and that the patients with an abnormal central pair show apparently less variability. Our study group included three such patients and PMN from one patient were analyzed on three different dates in the Neuro Probe chemotaxis assay and by flow cytometry. The results for this patient were congruent for the three testing dates.

### Chemoattractant Receptor Expression on PCD Neutrophils

**Figure 3:**
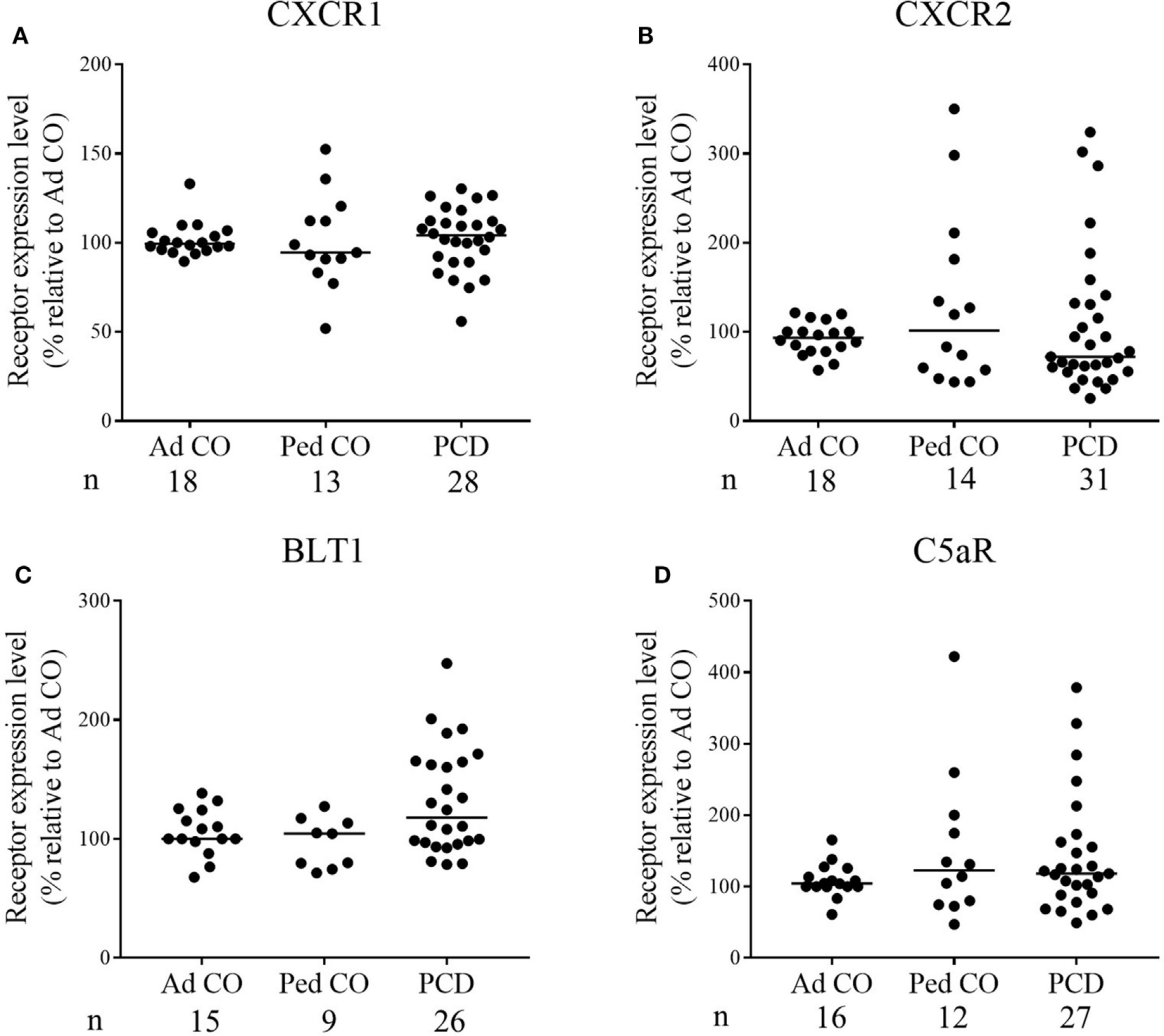
Chemoattractant receptor expression levels on PMN from patients with primary ciliary dyskinesia (PCD) and healthy controls. Expression levels of **(A)** CXCR1, **(B)** CXCR2, **(C)** BLT1 [the leukotriene B4 (LTB4) receptor], and **(D)** C5aR on PMN from patients with PCD, healthy children (Ped CO) and healthy adults (Ad CO) were measured by flow cytometry. The chemoattractant receptor expression levels [mean fluorescence intensity (MFI)] were normalized to the levels on PMN from the reference Ad CO (%). Each dot represents the normalized MFI of one patient or healthy control and the horizontal lines indicate the median value. The number of blood donors (*n*) in each experimental group is indicated below the *x*-axis. No statistical differences in chemoattractant receptor expression levels on PMN from patients with PCD and healthy controls were detected (Mann–Whitney *U* test).

Expression levels of CXCR1 (CXCL8 receptor) and CXCR2 (CXCL5 and CXCL8 receptor) on PCD PMN and Ad CO, measured by flow cytometry, did not significantly differ (Figures 3A,B); CXCR2 levels on PCD PMN only tended to be lower (median relative expression level 72%; *p* = 0.3383 PCD versus Ad CO). CXCR2 was downregulated in about 65% of the PCD patients. We performed a chi-square test to evaluate whether the proportion of individuals with a reduced CXCR2 expression differed between the PCD patients, on the one hand, and the healthy Ad CO or Ped CO, on the other hand. We defined three categories: <90, 90–110, and >110% CXCR2 expression level (relative to the reference Ad CO) and tested whether PCD patients and Ad COs were differently distributed (*p* = 0.11, chi-square test). Non-normalized CXCR2 flow cytometry profiles of neutrophils are shown in Figure S2 in Supplementary Material. Expression levels of the C5a and LTB4 receptors, BLT1 and C5aR, were not statistically different (Figures 3C,D). We explored a possible correlation between CXCR2 expression and the clinical parameter forced expiratory volume in 1 s (FEV_1_) and CXCR2 expression and the clinical parameter forced vital capacity (FVC) of the patients. However, we found no correlation between these lung function parameters and CXCR2 expression, but FEV_1_ correlated with the chemotactic response to CXCL8 (Pearson correlation coefficient *r* = 0.43, *p* = 0.02) (Figure S3 in Supplementary Material). Finally, we did not detect a clear effect of chronic infection or drug treatment on CXCR2 expression levels or CXCL8 response (data not shown). However, there was a big variability in disease severity, type of chronic infection, and treatment between the included patients.

### Confirmation of Reduced PCD PMN Responses to CXCL5 and CXCL8 in Alternative Activation Assays

**Figure 4:**
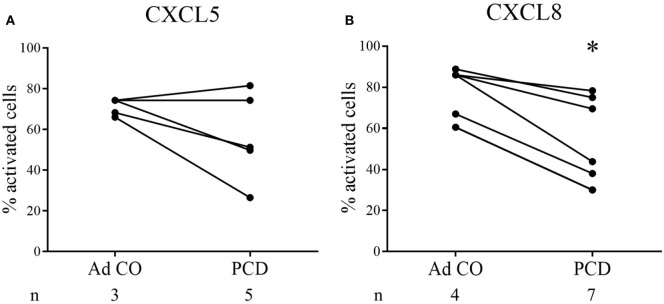
PMN from patients with primary ciliary dyskinesia (PCD) show reduced activation in response to CXCL8 in the shape change assay. In the shape change assay, PMN from patients with PCD and adult controls (Ad CO) were activated in suspension by addition of **(A)** CXCL5 (50 ng/ml) or **(B)** CXCL8 (50 ng/ml) for 1 min. The changes in cell shape were evaluated microscopically and the percentage of activated cells (showing an irregular outline with protrusions) was determined. The solid lines connect the corresponding patient and adult control within one experiment. The number of blood donors (*n*) in each experimental group is indicated below the *x*-axis. Statistical data analysis was performed using the Mann–Whitney *U* test (Ad CO versus PCD: *p < 0.05).

Subsequently, we microscopically analyzed changes in cell shape 1 min after stimulation of PCD PMN with CXCL5 or CXCL8. Figures 4A,B show the response to CXCL5 (*n* = 5) and CXCL8 (*n* = 7), respectively. Three out of five patients displayed a reduced response to CXCL5, whereas the PMN from all patients tested were less responsive to CXCL8 (*p* = 0.0478).

**Figure 5:**
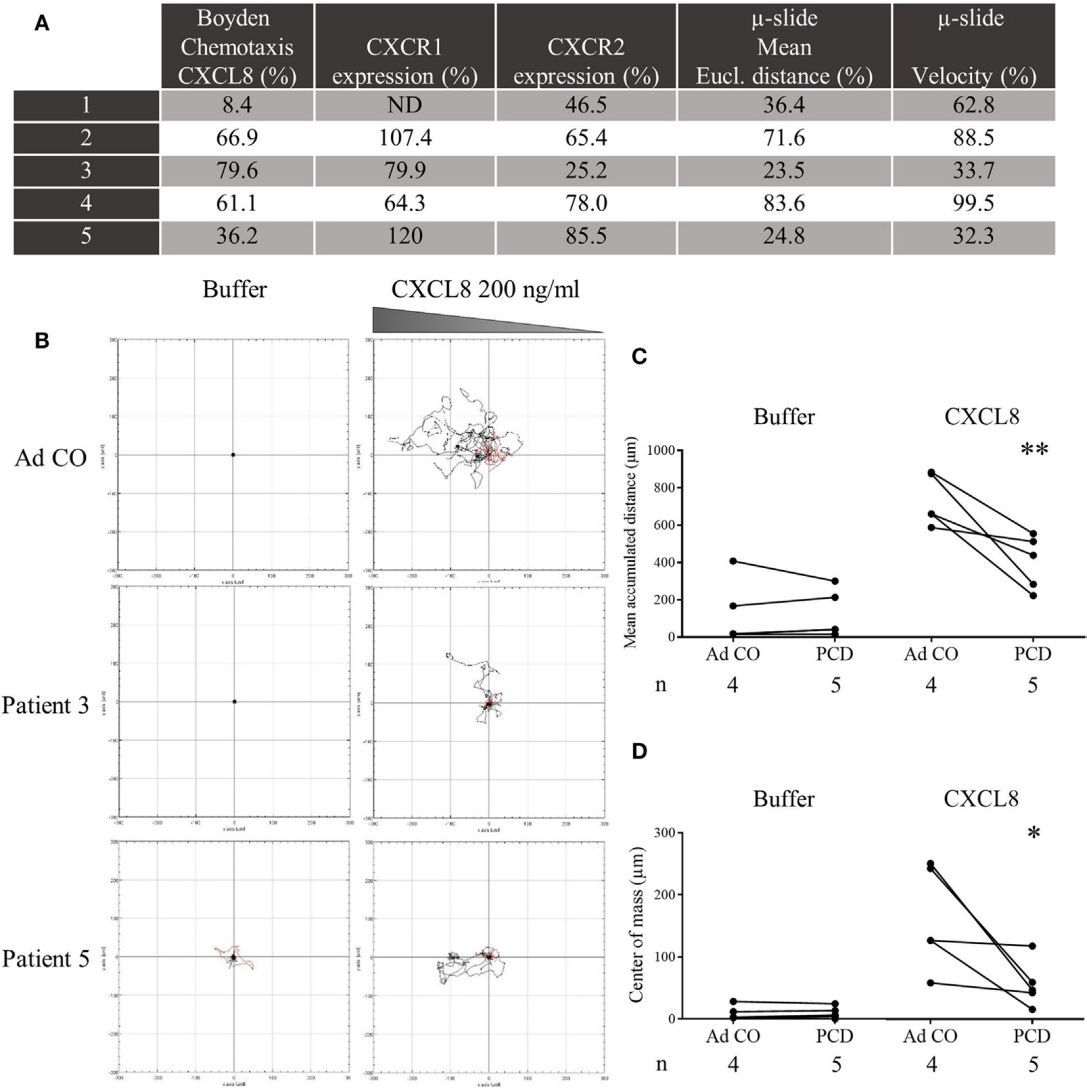
Decreased chemotactic capacity of peripheral blood PMN from patients with primary ciliary dyskinesia (PCD) to CXCL8 in the μ-slide chemotaxis assay. PMN from five patients with PCD were added to μ-slide chemotaxis chambers to assess their migratory capacity toward CXCL8 (200 ng/ml) in comparison to PMN from a healthy adult individual (Ad CO = 100%). Per condition, trajectory paths from 20 randomly picked cells were analyzed and several parameters were calculated. **(A)** On the same day, the PMN were tested in the Neuro Probe chemotaxis assay and analyzed by flow cytometry for CXCR1 and CXCR2 expression. The mean Euclidian distance and velocity of PCD PMN, measured in the μ-slide chemotaxis assay, were normalized to the mean Euclidian distance and velocity of PMN from the reference adult control (%). These parameters were significantly lower compared to the Ad CO (*p* < 0.01). **(B)** Representative cell tracks of two patients and an adult control are shown. For each experiment, **(C)** the mean accumulated distance or **(D)** center of mass obtained for the patient and adult control are connected by a solid line. The number of blood donors (*n*) in each experimental group is indicated below the *x*-axis. Statistical data analysis was performed using the Mann-Whitney *U* test (*p < 0.05; **p < 0.01) (*ND* = *not determined*).

In a second round of experiments using time-lapse microscopy, we monitored during 120 min migration of PMN from patients that showed a reduced chemotactic response to CXCL8 in the Neuro Probe chemotaxis chamber. The Neuro Probe chemotaxis assay and flow cytometry (CXCR1/2 expression) were repeated in parallel with the μ-slide chemotaxis assay (Figure 5A). We observed that PCD PMN migrated more slowly and, therefore, covered shorter distances toward CXCL8 than Ad CO PMN (Figures 5B–D). The directionality of migration was comparable for control and PCD PMN (data not shown). The decreased chemotactic response was, however, not associated with defective spontaneous migration, because the mean accumulated distance in response to buffer stimulation was similar for control and PCD PMN, confirming published findings (7).

### PBMCs from Patients with PCD Produce More CXCL8 and IL-1β in Response to Stimulation with Bacterial Products

**Figure 6:**
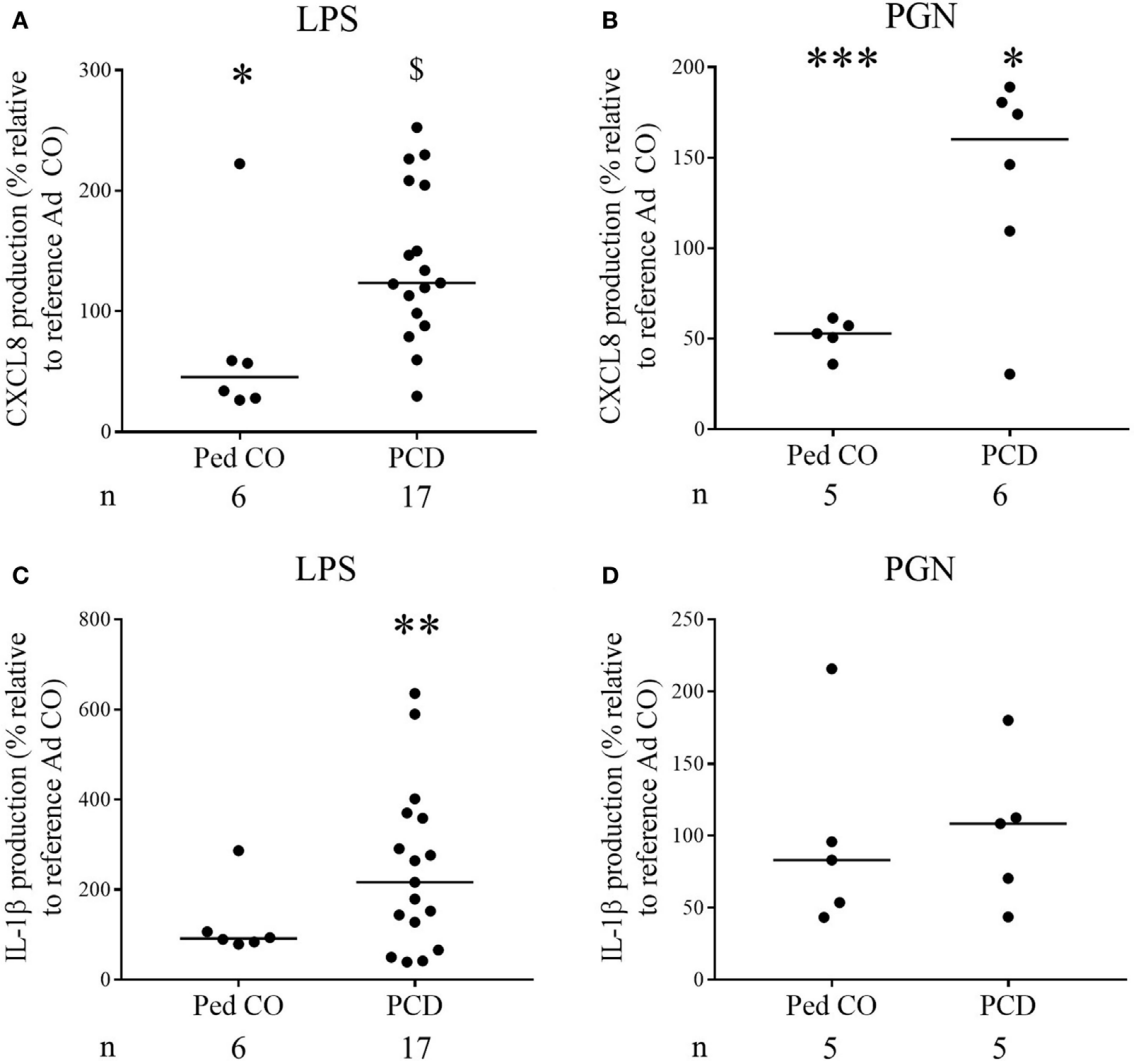
Increased production of CXCL8 by peripheral blood mononuclear cells (PBMCs) from PCD patients in response to pro-inflammatory stimuli. PBMCs from patients with PCD, healthy children (Ped CO) and healthy adults (Ad CO) were cultured and stimulated with **(A,C)** lipopolysaccharide (LPS) (500 ng/ml) and **(B,D)** PGN (10 µg/ml) at 37°C for 24 h. By sandwich ELISA, **(A,B)** CXCL8 and **(C,D)** IL-1β levels were measured in the supernatants. The CXCL8 and IL-1β levels are expressed relative to the CXCL8 and IL-1β levels of the reference Ad CO (equal to 100%, not shown). Each dot represents a separate blood donor and the horizontal lines indicate the median value. The number of blood donors (*n*) in each experimental group is indicated below the *x*-axis. Statistical data analysis was performed using the Mann–Whitney *U* test (Ad CO versus Ped CO or PCD: *p < 0.05, ***p < 0.001; Ped CO versus PCD: $ *p* < 0.05).

Finally, it is known that CXCL8 levels in PCD sputum are increased (3, 15). To test the hypothesis that this is due to overproduction of CXCL8 by PCD monocytes, we stimulated PCD PBMCs with several inflammatory mediators. The relative distribution of monocytes and lymphocytes in the PBMC fraction was similar in patients compared to healthy controls. On average, 9.7 ± 0.8% (mean ± SEM) CD14+ cells were present in PCD patients compared to 9.1 ± 0.6% (mean ± SEM) CD14+ cells in the Ad CO (*p* = 0.6933). All inducers tested stimulated production of CXCL8 by monocytes from healthy controls and patients with PCD (Figures 6A,B). Exact, non-normalized cytokine levels are shown in Table 2. The highest production of CXCL8 (402 ng/ml) was induced by LPS in PCD PBMCs. CXCL8 production after LPS treatment was significantly (*p* < 0.05) enhanced in PCD compared to Ped CO (Figure 6A). Also PGN provoked higher CXCL8 production by PCD monocytes compared to monocytes of Ad CO (323 and 260 ng/ml, respectively, *p* < 0.05), however, due to the number of individuals tested, this difference was not statistically significantly for PCD versus Ped CO (*p* = 0.0823) (Figure 6B). IL-1β stimulation also led to higher CXCL8 production by PCD monocytes compared to both healthy control groups (75, 25, and 15 ng/ml, respectively; not shown). Since IL-1β is an important CXCL8 inducer (16), we also measured IL-1β in the PBMC supernatants (Figures 6C,D). IL-1β production was higher in PCD compared to Ad CO upon LPS stimulation (16 and 7 pg/ml for PCD and Ad Co, respectively; *p* < 0.01) (Figure 6C), but not upon PGN stimulation (Figure 6D). Thus, PCD monocytes produce more CXCL8 and IL-1β in inflammatory conditions and IL-1β provoked higher CXCL8 production by PCD monocytes in turn. The increased CXCL8 production can downregulate CXCR2 expression and cause CXCR2 desensitization on PMN, providing an explanation for the reduced migration toward CXCL8. We must admit that not all patients showed reduced CXCR2 levels. However, it has been shown that in some circumstances chemokine receptors, although being upregulated, transmit migratory responses less efficiently (17–19).

**Table 2:** The non-normalized CXCL8 and IL-1β levels released by peripheral blood mononuclear cells (PBMCs) from adult controls (Ad CO), pediatric controls (Ped CO) and patients with primary ciliary dyskinesia (PCD).

Cytokine measured	CXCL8 (mean ± SEM) (ng/ml)		IL-1β (mean ± SEM) (pg/ml)	
Stimulus	LPS	PGN	LPS	PGN
Ad CO	175.4 ± 22.8	246.6 ± 27.5	7.4 ± 1.5	11.6 ± 2.7
Ped CO	123.9 ± 34.8	139.4 ± 16.3	11.1 ± 2.4	7.7 ± 1.8
PCD	203.0 ± 27.9	390.4 ± 60.9	15.7 ± 4.9	11.1 ± 2.7

*LPS, lipopolysaccharide; PGN, peptidoglycan*.

*PBMCs were cultured and stimulated with LPS (500 ng/ml) or PGN (10 µg/ml) at 37°C for 24 h. By sandwich ELISA, CXCL8, and IL-1β levels were measured in the supernatants*.

## Discussion

Our study of the chemotactic response of PCD neutrophils to four chemoattractants, C5a, LTB4, and the chemokines CXCL5 and CXCL8, can be motivated by multiple arguments. First, there is no consensus whether PCD neutrophils are affected in their migratory response, because both normal (8, 9) and decreased (6, 7) migration of PCD neutrophils has been reported in the past. Of note, chemokines were not included in those studies. In addition, it is known that chronic inflammation influences functioning of neutrophilic granulocytes in cystic fibrosis (CF) and chronic obstructive pulmonary disease (COPD). PCD and CF, though caused by completely different gene defects, share some phenotypical characteristics, i.e., an important neutrophilic infiltrate being present in the lungs, impaired mucociliary clearance and recurrent airway infections. It has been shown that neutrophil survival, metabolism, effector, and regulatory functions are affected in CF due to mutations in the *CFTR* gene, but also as a consequence of chronic inflammation (20). We here demonstrate that also PCD neutrophils display aberrant chemotactic activity. We observed that in contrast to normal migration to LTB4 and C5a, chemoattractants acting early in the extravasation process, we observed reduced migration toward the CXCR2 ligands CXCL5 and CXCL8, which act later on in the cascade guiding neutrophils from the blood stream to the inflamed tissue (21). The importance of CXCR2 in pathological PMN recruitment to inflamed lungs is underlined by the extensive efforts put into development of CXCR2 antagonists as a therapeutic approach to prevent lung injury by neutrophils in, e.g., COPD and CF (22, 23). Indeed, during severe exacerbations of COPD, enhanced levels of CXCR2 mRNA were detected correlating with the presence of tissue neutrophils (24). Furthermore, acute lung injury patients with CXCL8 gene polymorphisms expressing higher levels of CXCL8 have more prolonged and extensive lung injury (25). On the other hand, it is long known that neutrophils are needed to efficiently cope with bacterial infections (26) and CXCL5 was indispensable in several mouse models of infection (27, 28). Thus, the recruitment and activation of neutrophils in the lungs must be well-balanced and tightly regulated. Finally, also CC chemokines contribute to neutrophil recruitment to injured lungs (26). A recent study reported that in acute respiratory distress syndrome (ARDS), CCL2, and CCL7 synergize with CXCL8 to promote neutrophil migration (29).

Today, about 35 genes are known to be mutated in PCD. Because neutrophils and leukocytes in general do not have motile cilia, only some of these genes (which have a role in the building-up of motile cilia in the cytoplasm) are expressed in leukocytes. The genes mutated in the genotyped patients with PCD included in this study are not expressed in neutrophils. However, the genetic defect is not known in one-third of the included patients (Table 1). Therefore, we cannot rule out that in some patients chemotaxis is reduced as a primary consequence of the mutation in PCD genes, whereas in others the defect is a secondary effect due to chronic infection/inflammation.

To reveal the molecular cause of reduced responsiveness of PCD PMN to CXCL5 and CXCL8, we determined expression levels of the CXC chemokine receptors involved (CXCR2 for CXCL5 and CXCR1 and CXCR2 for CXCL8). CXCR1 levels were normal, but in many patients CXCR2 levels were reduced. Several mediators, such as TLR2 and TLR4 ligands and cytokines, are known to regulate CXCR2 expression on neutrophils with nitric oxide and G protein-coupled receptor kinases (GRKs) acting downstream (30–32). In sepsis patients, LPS induces expression of GRK2 and downregulation of CXCR2 (33). LPS-induced CXCR2 downregulation is blocked in phospholipase D2 knock-out mice (34) demonstrating involvement of this phospholipase upstream of GRK2. Although some similarities between neutrophils from PCD and sepsis patients might be noticed, LTB4 and fMLP responses in patients with sepsis were also reduced (31). To assess whether altered chemotaxis responses were related to changes in granulopoiesis or in the maturity of circulating neutrophils, we analyzed CD16 and CD11b expression. We found no significant differences in expression of both markers between the adult controls and the patients with PCD (CD16 expression (mean ± SEM) on neutrophils of PCD relative to Ad CO: 114.0 ± 9.8%, *p* = 0.1968; CD11b expression (mean ± SEM) on neutrophils of PCD relative to Ad CO: 152.4 ± 25.3%, *p* = 0.3890).

Remarkably, the expression levels of CXCR2 varied greatly between the studied patients (Figure 3), though the reduced response to CXCL8 was observed more consistently. Upregulation of CXCR2 in some patients, versus downregulation in others, might relate to different degree of pathology or stage of disease or to a different pulmonary microenvironment. However, increased chemokine receptor expression does not exclude reduced responsiveness. Indeed, enhanced levels of CCR1 and CCR5 were shown to be present on monocytes from hemolytic uremic syndrome (HUS) patients, but HUS monocytes did not respond as well as monocytes from healthy individuals in functional assays (18). Also in patients with COPD, more monocytes expressed CCR5 as a result of enhanced IL-6/sIL-6R expression, whereas less monocytes migrated toward sputum supernatant compared to non-smokers (19). D’Amico *et al*. demonstrated that IL-10 treatment upregulates chemokine receptors on monocytes, but those receptors are not coupled anymore to the classic signal transduction pathways and act as functional decoys (35).

One could argue that this study is lacking a disease control to examine whether our findings are specific for PCD. Pediatric patients with CF are not suited as disease control, because mutations in the *CFTR* gene disturb neutrophil function (5). Patients with non-CF bronchiectasis also establish neutrophilic airway disease, but this group is so heterogeneous and some patients with unknown underlying immune deficiency could in this way be included. Asthma is rather an eosinophilic disorder, and has a completely different pathophysiology. Therefore, we believe that inclusion of an appropriate disease control group is not feasible. Moreover, our experimental set-up (chemotaxis assays) is very labor intensive and only a limited number of donors can be processed at a time.

Our results suggest that in patients with PCD decreased migration of peripheral blood PMN toward CXCL5 and CXCL8 might contribute to inefficient clearance of pathogens from the airways. It is unclear whether this defect is primary or acquired. The defective response might be caused by desensitized CXCR2 receptors due to constantly enhanced CXCL8 production in the airways. Indeed, we have shown here that PCD monocytes produce more CXCL8 and IL-1β, an important CXCL8 inducer, than healthy monocytes in response to inflammatory triggers. In addition, increased CXCL8 levels were reported in the sputum of patients with PCD (3, 15). On the other hand, since there is no apparent correlation between defective migration and type or severity of lung infection and inflammation (as expressed by age, infecting pathogen, severity of lung function, or structural lung disease as assessed by imaging; see Table 1), we cannot exclude an inherent PMN defect.

## Ethics Statement

This study was carried out in accordance with the study protocol (S57236[ML11095]) that was approved by the ethical committee of the University hospital of Leuven/KU Leuven with written informed consent from all subjects. All subjects gave written informed consent in accordance with the Declaration of Helsinki.

## Author Contributions

KDB, JVD, MB, and SS designed the study; MC and SS wrote the paper. All authors analyzed and interpreted the data. VG, KDB, and MB recruited pediatric patients with PCD. MC, MG, and SS performed experiments. All authors revised the work and approved the version to be published.

## Conflict of Interest Statement

The authors declare that the research was conducted in the absence of any commercial or financial relationships that could be construed as a potential conflict of interest.
