# Supplementary material for: Neutrophils from Patients with Primary Ciliary Dyskinesia Display Reduced Chemotaxis to CXCR2 Ligands
Source: Front Immunol. 2017 Sep 22;8:1126. doi: 10.3389/fimmu.2017.01126 (PMC5614927; doi:10.3389/fimmu.2017.01126)
Supplement: Supplementary file 1 [file data_sheet_1.pdf]

Supplementary Material

Neutrophils from patients with primary ciliary dyskinesia display reduced chemotaxis to CXCR2 ligands

Maaike Cockx[1], Mieke Gouwy[1], PhD, Véronique Godding[2], MD, Kris De Boeck[3], MD PhD, Jo Van Damme[1], PhD, Mieke Boon[3], MD PhD, Sofie Struyf[1*], PhD

**Corresponding author*:
E-mail: sofie.struyf@kuleuven.be
Tel: +32 16 32 24 22
Address: Rega - Herestraat 49 - bus 1042 - 3000 Leuven

2 Supplementary Figures

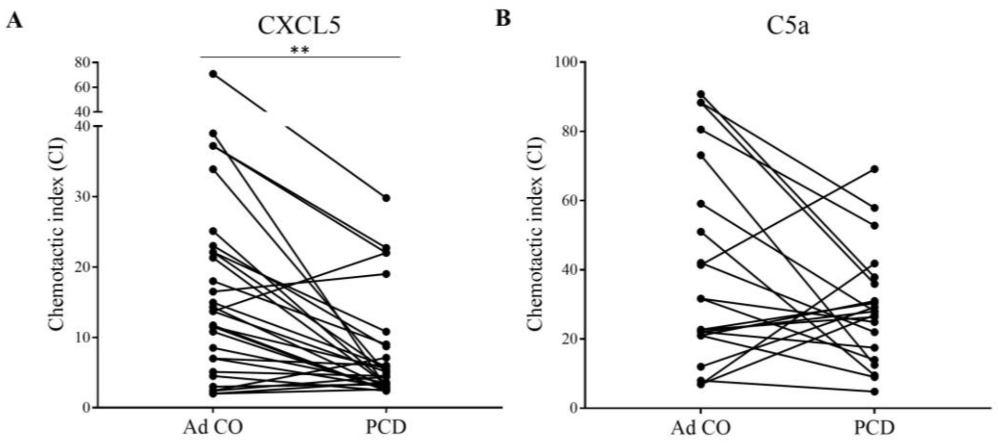

Supplementary Figure 1. Chemotactic indices (CI) of neutrophils from patients with PCD in response to CXCL5 and C5a
After having counted the cells that migrated in the Neuro Probe chemotaxis chamber, the chemotactic index (CI) is calculated as the average number of migrated cells in response to the chemokine divided by the average spontaneously migrated cells in buffer conditions. The CI gives a quantitative measurement of how much more cells migrated in response to the chemokine compared to buffer conditions. The calculated CIs of neutrophils from patients with PCD (PCD) and adult controls (Ad CO) in response to (**A**) CXCL5 (10 ng/ml) and (**B**) C5a (10 ng/ml) are displayed. The solid lines connect the CI value of the patient with the CI value of its reference Ad CO. A significantly lower response to CXCL5 of neutrophils from patients with PCD compared to Ad CO was demonstrated (**p<0.01; Mann Whitney U-test), whereas no difference in chemotactic migration to C5a was measured.

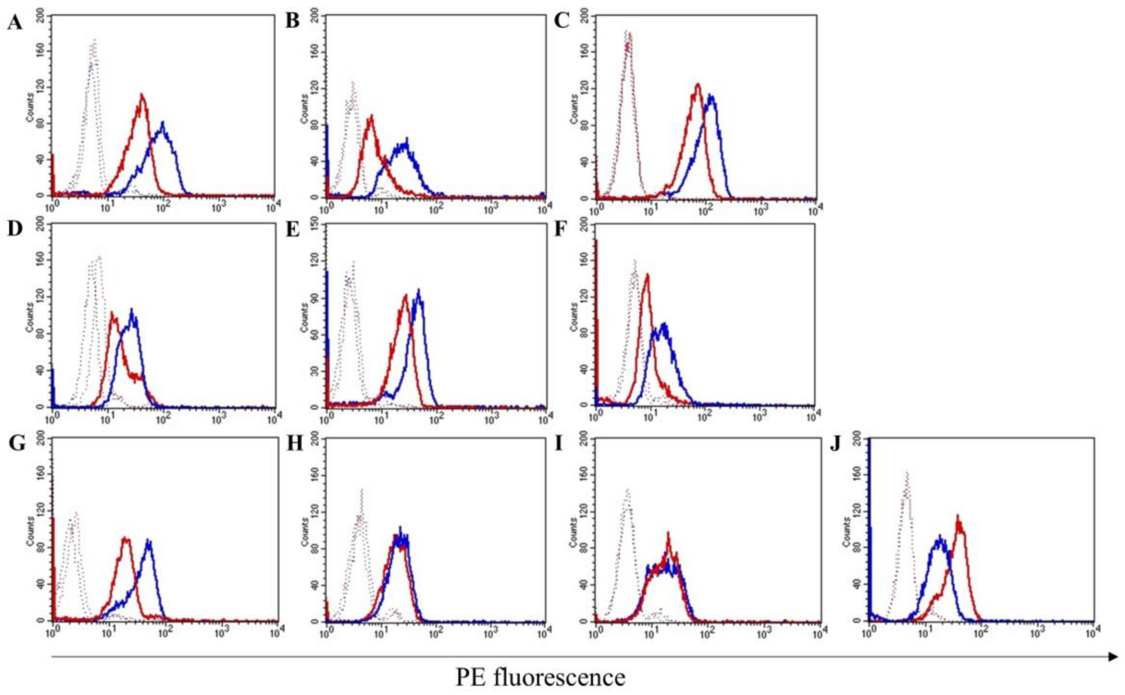

Supplementary Figure 2. Flow cytometric profiles of CXCR2 expression on neutrophils from patients with PCD

Flow cytometry was used to measure the expression of CXCR2 on neutrophils from patients with PCD and adult controls (Ad COs). On each figure, the dotted lines are the fluorescence profiles of neutrophils labeled with PE-labeled secondary antibody only (negative control), the continuous lines represent the fluorescent profiles of neutrophils labeled with the primary CXCR2 antibody combined with PE-labeled secondary antibody. The red lines represent the fluorescence profile of neutrophils from the patient, the blue lines that from the reference Ad CO. Ten representative histograms from patients with PCD are displayed: (**A-G**) seven patients with lower CXCR2 expression compared to the accompanying Ad CO, (**H,I**) two patients with equal CXCR2 expression compared to the accompanying Ad CO and (**J**) one patient with increased CXCR2 expression.

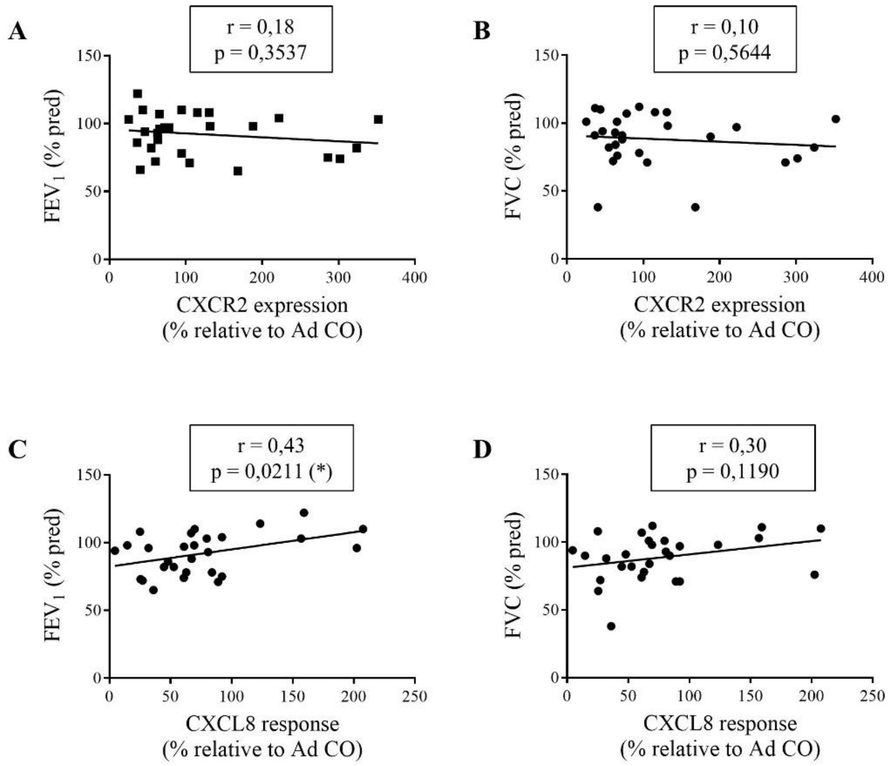

Supplementary Figure 3. Correlation between CXCR2 expression or CXCL8 response and lung function in the studied patients with PCD

CXCR2 expression on neutrophils from the studied patients with PCD [relative to the CXCR2 expression on neutrophils from the reference adult control (Ad CO)] is plotted against the clinical parameters (**A**) FEV_1 (% pred) and (**B**) FVC (% pred). Also, the neutrophil chemotactic response to CXCL8 in the Neuro Probe chemotaxis assay (relative to the CXCL8 response of neutrophils from the reference Ad CO) is plotted against (**C**) FEV_1 (% pred) and (**D**) FVC (% pred). A possible correlation was tested by fitting a linear regression curve. (Pearson correlation coefficient analysis)

FEV_1 (% pred): forced expired volume in 1 second (predicted %); FVC (% pred): forced vital capacity (predicted %)
